# Stability Boundary and Enhanced Solution of Dual-Mode Based Micro Gyroscope Mode Matching Technology

**DOI:** 10.3390/mi13081251

**Published:** 2022-08-03

**Authors:** Changda Xing, Xinning Wang, Zishuo Wang, Yuchen Wang, Chong Li

**Affiliations:** 1 School of Engineering, Ocean University of China, Qingdao 266102, China; xing.changda@foxmail.com (C.X.); 17806259460@163.com (Z.W.); wyc@stu.ouc.edu.cn (Y.W.); czl0047@auburn.edu (C.L.); 2 Teaching Center of Fundamental Courses, Ocean University of China, Qingdao 266102, China

**Keywords:** MEMS gyroscope, dual-mode scheme, mode matching, mode switching

## Abstract

During in-run mode matching under a dual-mode gyro scheme, the stability of the closed-loop control system has a boundary. This phenomenon will lead to the failure of the in-run frequency split calibration scheme when the initial mode mismatch is too severe to exceed the stability boundary. This paper gives a detailed analysis of this stability boundary through simulations and experiments. Results show that the length of the stable region will be affected by the resonant frequency and the *Q* value. High resonant frequency and low *Q* value will widen the stable region, but also reduce the sensitivity and rapidity of the calibration. In order to remove the limitation of the stability boundary while applying the in-run frequency split calibration under dual-mode architecture, this paper proposes an enhanced solution that combines both the dual-mode scheme and technology of mode switching. The application of mode switching achieves a pre-calibration of frequency split before the normal gyro operation. This solution is implemented in engineering on a hybrid gyro interface circuit prototype with single-mode and dual-mode. Validation experiments confirmed the effectiveness of this solution.

## 1. Introduction

A MEMS (Micro-Electro-Mechanical System) gyroscope is a type of inertial sensor that measures angular rate based on the Coriolis effect [1,2]. It has the advantages of small size, light weight, low power consumption, and low price [3]. Nowadays, MEMS gyroscopes have been widely used in micro-inertial navigation, industrial control, and consumer electronics, etc. [4,5,6]. In order to improve the sensitivity of angular rate detection, MEMS gyroscopes are generally developed in the direction of mode matched and high-quality factors (*Q*) [7]. However, this scheme has very high requirements for the matching condition between the gyroscopic modes, because gyro with high *Q* value will be extremely sensitive to mode mismatch [8].

Under the existing MEMS gyroscope fabrication technology, the matching condition between the gyroscopic modes is usually unsatisfactory [9]. This means that mode mismatch will inevitably appear among gyroscopic modes. Mode mismatch will reduce the sensitivity of signal detection and the reliability of the scale factor [10]. Additionally, this error will cause the bias drift of gyro, resulting in poor bias stability and repeatability [11,12]. In order to make the gyro work properly, effective mode matching technology is essential.

At present, a lot of research works have been carried out on the calibration of mode mismatch. These works can be grouped into three types. The first type is to optimize the structural design and fabricating technology of the gyro [13,14,15]. For example, Bo Fan’s group designed a novel cobweb-like disk resonator gyroscope in 2019 [8]. Owing to the all-linear structure, this type of gyro has fewer fabrication imperfections. The smallest as-fabricated relative frequency split (29.9 ppm) of this cobweb-like disk resonator gyroscope was about 10.8 times smaller than that (322.5 ppm) of the ring-like disk resonator gyroscope. The second type is to perform a post-trimming to the gyro [16,17]. In 2018, Yuting Wang from the National University of Defense Technology decreased frequency splits of hemispherical resonators by chemical etching [18]. This work solves the inconsistency of traditional laser trimming for fused silica materials. However, this kind of method may only be performed pre-packaging and can cause permanent damage to the structure. The third type is to suppress the frequency split by electrostatic tuning [19,20], which has been widely used in MEMS gyros because of its simplicity and effectiveness. The main advantages of this type of method are the accuracy of tuning and the convenience to implement digitally [21]. The work of Zhongxu Hu et al. in 2011 used simplified displacement feedback in a high-performance DSP-based rate gyroscope control system to reduce the frequency split between gyroscopic modes [22]. Experimental results showed that the frequency split is suppressed to 0.01 Hz. However, with the change of the environmental parameters, the mode mismatch will become worse [23,24]. This means that in the actual operation of gyro, the calibration of frequency split has in-run requirements [25], which illustrates the need for an advanced in-run mode matching method.

Arashk Norouzpour-Shirazi et al. put forward a novel dual-mode actuation and sensing scheme for read-out and calibration of axisymmetric Coriolis resonant gyroscopes [26]. The core idea is to actuate both modes of an axisymmetric gyroscope with two identical in-phase excitation signals, and sense both modes concurrently. This scheme utilizes the sum and difference of the sense signals to demonstrate complete cancellation of the gyroscope bias terms, and provide automatic in-run mode matching capability.

However, for now, explorations of this dual-mode scheme only focused on its accuracy of mode matching, while the stable range of the closed-loop control system is still unknown. If the initial frequency split caused by mode mismatch exceeds the stable boundary, the mode matching method based on dual-mode scheme may fail. This will bring difficulties to the application of dual-mode scheme. Therefore, it is necessary to explore the properties of this stability boundary and propose effective solutions to eliminate the limitation brought by stability boundary.

Contributions of this work are summarized as follows:

(1) Based on the in-run mode matching under dual-mode scheme, the stability boundary of the initial frequency split is analyzed theoretically and experimentally;

(2) To eliminate the limitation brought by stability boundary, an enhanced solution is proposed and implemented in engineering;

(3) Validation experiments are performed on a prototype. The experimental results show the effectiveness of the proposed scheme.

## 2. Theoretical Modeling and Simulation

### 2.1. Gyroscope Dynamics under Dual-Mode Scheme

The dynamics of the MEMS gyroscope can be described by the following set of equations [27]:

(1) $$\begin{array}{c} \begin{array}{cc} \; & m\ddot{x}+c_{x}\dot{x}+k_{x}x+c_{yx}\dot{y}+k_{yx}y=F_{x}-2m\lambda \Omega_{z}\dot{y}\\ \; & m\ddot{y}+c_{y}\dot{y}+k_{y}y+c_{xy}\dot{x}+k_{xy}x=F_{y}+2m\lambda \Omega_{z}\dot{x}, \end{array} \end{array}$$

where *x* and *y* are the generalized coordinates, *m* is the proof mass, $k_{x}$ and $k_{y}$ are the sprint stiffness of the two modes, $c_{x}$ and $c_{y}$ are the damping coefficients of each mode, $k_{xy}$ and $k_{yx}$ are the stiffness coupling coefficients between the two modes, $c_{xy}$ and $c_{yx}$ are the damping coupling coefficients between the two modes, $F_{x}$ and $F_{y}$ are the driven forces, λ is the Coriolis angle gain determined by the mechanical structure design and the modal order, and $\Omega_{z}$ is the rotation rate in the Z axis.

In practice, the following quantities are commonly used:

(2) $$\begin{array}{c} \begin{array}{cc} \; & \omega_{x}=\sqrt{k_{x}/m},\;\;\;\;c_{x}/m=2\zeta_{x}\omega_{x}=\omega_{x}/Q_{x},\\ \; & \omega_{y}=\sqrt{k_{y}/m},\;\;\;\;c_{y}/m=2\zeta_{y}\omega_{y}=\omega_{x}/Q_{y},\\ \; & \omega_{xy}=\sqrt{k_{xy}/m},\;c_{xy}/m=\rho_{xy}, \end{array} \end{array}$$

where $\omega_{x}$ and $\omega_{y}$ are the resonant frequencies of the two modes, $Q_{x}$ and $Q_{y}$ are the quality factors of the two modes, $\omega_{xy}$ is the cross-coupling frequency term, $\rho_{xy}$ is the cross-coupling damping term, and $\zeta_{x}$ and $\zeta_{y}$ are the damping ratios.

**Figure 1:**
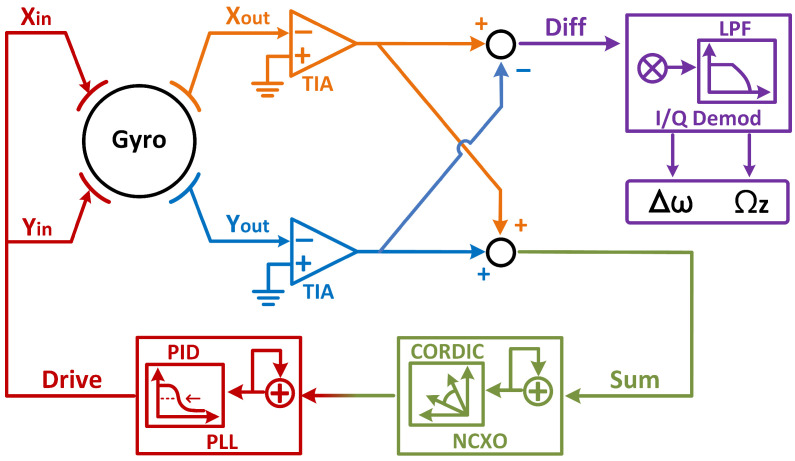
The schematic of the dual-mode gyroscope architecture.

The architecture of dual-mode actuation and sensing scheme is shown in Figure 1. Under dual-mode architecture, both modes of the gyro are actuated and sensed simultaneously [11]. The sum signal is used for self-sustaining closed-loop actuation of the gyroscope, and the difference signal is used for the detection of Coriolis signal. Assuming that the two modes of the gyro are symmetric, the two gyroscopic modes of the device are driven simultaneously by:

(3) $$\begin{array}{c} \begin{array}{cc} \; & F_{x}=F_{y}=F_{0}\cos\left(\omega_{0}t\right), \end{array} \end{array}$$

where $\omega_{0}=\left(\omega_{x}+\omega_{y}\right)/2$. In this case, it can be considered that $Q_{x}\approx Q_{y}=Q_{0}$ for the two gyroscopic modes.

Using the Laplace transform technique to analyze the response of (1), the sum and difference of the two modes’ outputs can be derived as:

(4) $$\begin{array}{cc} \; & Z_{\mathrm{sum}\;}\left(j\omega_{0}\right)\\ \; & =X\left(j\omega_{0}\right)+Y\left(j\omega_{0}\right)\\ \; & =-\frac{2F_{0}}{m\omega_{0}}\frac{{\left(\Delta \omega \right)}^{2}/4\omega_{0}-\omega_{xy}^{2}/\omega +j\omega_{0}/Q_{0}-j\rho_{xy}}{\left[\begin{array}{c} {\left(\Delta \omega \right)}^{2}+{\left(\frac{\omega_{0}}{Q_{0}}\right)}^{2}+{\left(2\lambda \Omega_{z}\right)}^{2}-\rho_{xy}^{2}+\frac{\omega_{xy}^{4}}{\omega_{0}^{2}}\\ +j\left(\frac{{\left(\Delta \omega \right)}^{2}}{2Q_{0}}+\frac{2\rho_{xy}\omega_{xy}^{2}}{\omega_{0}}\right)-\frac{\Delta \omega^{4}}{16\omega_{0}^{2}}-\frac{\Delta \omega^{2}}{4Q_{0}^{2}} \end{array}\right]}\\ \; & Z_{\mathrm{diff}\;}\left(j\omega_{0}\right)\\ \; & =X\left(j\omega_{0}\right)-Y\left(j\omega_{0}\right)\\ \; & =-\frac{2F_{0}}{m\omega_{0}}\frac{\Delta \omega +\Delta \omega /2Q_{0}+j2\lambda \Omega_{z}}{\left[\begin{array}{c} {\left(\Delta \omega \right)}^{2}+{\left(\frac{\omega_{0}}{Q_{0}}\right)}^{2}+{\left(2\lambda \Omega_{z}\right)}^{2}-\rho_{xy}^{2}+\frac{\omega_{xy}^{4}}{\omega_{0}^{2}}\\ +j\left(\frac{{\left(\Delta \omega \right)}^{2}}{2Q_{0}}+\frac{2\rho_{xy}\omega_{xy}^{2}}{\omega_{0}}\right)-\frac{\Delta \omega^{4}}{16\omega_{0}^{2}}-\frac{\Delta \omega^{2}}{4Q_{0}^{2}} \end{array}\right]}, \end{array}$$

where $\Delta \omega =\omega_{x}-\omega_{y}$ is the frequency split between the two gyroscopic modes. In the actual gyroscope, $\Delta \omega <<\omega_{0}$, and $Q_{0}<<1$. So the ${\left(\Delta \omega \right)}^{2}/2Q_{0}$ term in the above formulas can be ignored compared to ${\left(\omega_{0}/Q_{0}\right)}^{2}$. Similarly, the ${\left(\Delta \omega \right)}^{2}/2\omega_{0}$ term in the sum solution can also be ignored compared to $\omega_{0}/Q_{0}$. Therefore, the sum and difference solutions can be simplified as [27]:

(5) $$\begin{array}{cc} Z_{\mathrm{sum}\;}\left(j\omega_{0}\right) & =X\left(j\omega_{0}\right)+Y\left(j\omega_{0}\right)\\ \; & =-\frac{F_{0}}{m\omega_{0}}\frac{2j\omega_{0}/Q_{0}}{{\left(\Delta \omega \right)}^{2}+{\left(\frac{\omega_{0}}{Q_{0}}\right)}^{2}+{\left(2\lambda \Omega_{z}\right)}^{2}}\\ Z_{\mathrm{diff}\;}\left(j\omega_{0}\right) & =X\left(j\omega_{0}\right)-Y\left(j\omega_{0}\right)\\ \; & =-\frac{F_{0}}{m\omega_{0}}\frac{2\Delta \omega +4j\lambda \Omega_{z}}{{\left(\Delta \omega \right)}^{2}+{\left(\frac{\omega_{0}}{Q_{0}}\right)}^{2}+{\left(2\lambda \Omega_{z}\right)}^{2}}. \end{array}$$

It can be seen from (5) that the common-mode cross-coupling terms between the X and Y modes are completely cancelled out in the numerator of the difference signal $Z_{\mathrm{diff}\;}$. This makes dual-mode architecture a much better scheme on restraining the bias drift of gyro. In the imaginary part of $Z_{\mathrm{diff}\;}$, the differential Coriolis terms in the two sense outputs are added. So, the angular rate sensitivity is doubled. In the real part of $Z_{\mathrm{diff}\;}$, the difference signal is determined almost exclusively by the frequency split Δω. So the real-time frequency split between the two gyroscopic modes can be obtained by monitoring the real part of $Z_{\mathrm{diff}\;}$. Thus, by applying closed-loop control method, an in-run frequency split calibration algorithm can be implemented. Compared to the traditional offline calibration, the in-run calibration based on dual-mode architecture shows superiority of mode-matching speed, accuracy, and adaptability to the environmental changes.

### 2.2. Simulations of the Relationship between Δω and the Real Part of $Z_{\mathrm{diff}\;}$

Although the above in-run frequency split calibration scheme shows great advantages, there are still many details that need to be considered in practical applications. From the expression of difference signal in (5), it can be seen that the frequency split term Δω appears in both the numerator and denominator of the real part. Additionally, Δω has different orders in the numerator and denominator. This means that the relationship between frequency split and the real part of difference output is not purely linear. Moreover, in a wide range of Δω, the relationship curve between frequency split and the real part of difference output may not be monotonic. In this case, the closed-loop control system of frequency split will have stability boundary, which will lead to the failure of the in-run frequency split calibration scheme mentioned above.

In addition, it is worth noting that the expression of difference signal also includes the *Q* value term $Q_{0}$ and the resonant frequency term $\omega_{0}$. Although for a specific gyro, $Q_{0}$ and $\omega_{0}$ can be considered as constants. However, from the perspective of gyro manufacturing, different *Q* value and resonant frequency will inevitably affect the relationship between the frequency split and the difference signal. This will also affect the stability boundary of in-run frequency split calibration scheme.

**Figure 2:**
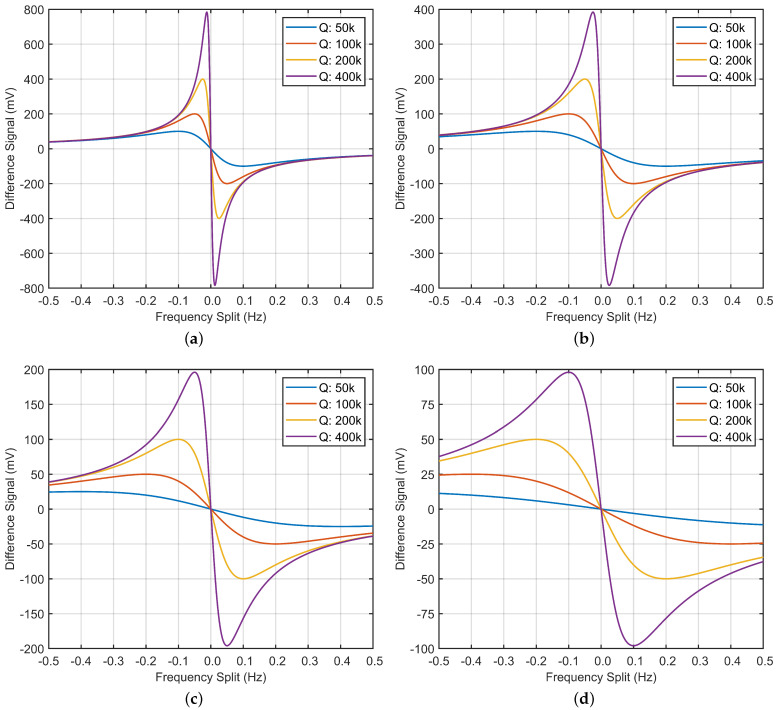
The relationship between the frequency split Δω and the real part of difference signal $Z_{\mathrm{diff}}$. (**a**) Resonant Frequency 5 kHz. (**b**) Resonant Frequency 10 kHz. (**c**) Resonant Frequency 20 kHz. (**d**) Resonant Frequency 40 kHz.

In order to have a comprehensive analysis of the relationship between the frequency split and the real part of difference output in (5), a MATLAB simulation with different *Q* values and resonant frequencies is implemented. The simulation results are shown in Figure 2.

From the simulation results above, it can be seen that the relationship between frequency split and the real part of difference signal is neither linear nor monotonic. When the frequency split is small, the relationship curve can be approximately considered to be linear. Additionally, when the frequency split Δω is zero, the real part of difference signal is also zero. Therefore, in this condition, the in-run frequency split calibration can be realized by applying closed-loop control method to keep the amplitude of the difference signal at zero. This is the theoretical basis of the frequency split calibration under dual-mode scheme. However, when the frequency split is large enough, the relationship curve enters the non-linear region. In this region, the monotonicity of the curve is opposite to the linear region. As the amplitude of the difference signal decreases, the frequency split between the two modes tends to increase. This means the effect of the closed-loop control system is no longer stable. Because of the stability boundaries caused by monotonicity change, the pure in-run frequency split calibration scheme is no longer applicable if the initial frequency split is too big.

Additionally, the resonant frequency and *Q* value have an important influence on the stability boundary of the in-run frequency split calibration scheme. It can be inferred from Figure 2 that higher resonant frequency can widen the stable region suitable for the in-run frequency split calibration. However, correspondingly, the sensitivity of the difference signal to the frequency split will decay, which may have a negative impact on the rapidity of the calibration. On the contrary, as the *Q* value increases, the stable region suitable for the in-run frequency split calibration will be shortened. At the same time, the sensitivity of the difference signal to the frequency split will rise, which may help to improve the rapidity of the calibration. All in all, the relationship curve between frequency split and the real part of difference signal is sensitive to both resonant frequency and *Q* value. This will further lead to the change of the stability boundary. If the in-run frequency split calibration is required, some trade-offs may be needed in the design of the gyro structure. So, the balance between stable calibration range and calibration rapidity can be achieved.

## 3. Enhanced Solution

From the analysis in Section 2, it can be known that the in-run frequency split calibration based on dual-mode scheme will fail when the initial frequency split exceeds the stability boundary. This will greatly limit the practical application of dual-mode scheme. Aiming at this problem, this section proposes an enhanced solution that combines both the dual-mode scheme and technology of mode switching.

### 3.1. Principle of Electrostatic Frequency Tuning

**Figure 3:**
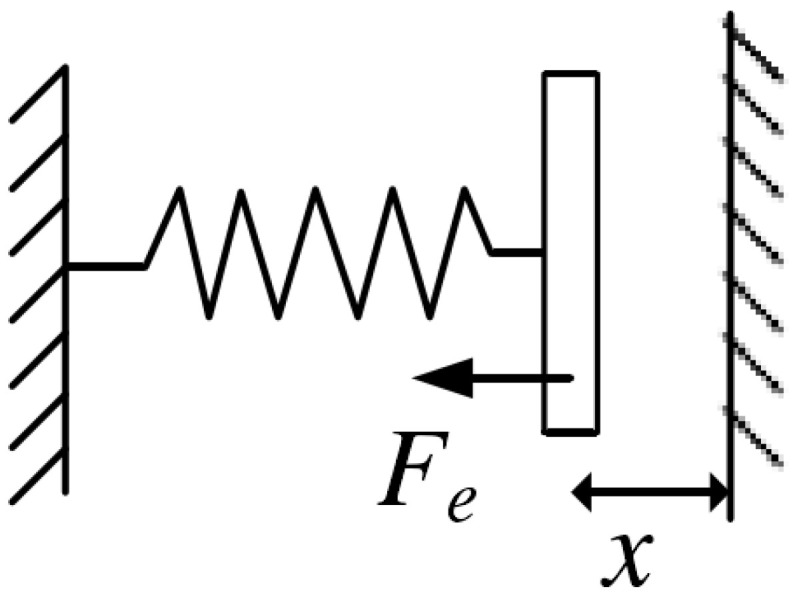
Simplified schematic of the tuning electrode.

Based on negative stiffness effect, electrostatic frequency tuning can change the equivalent stiffness of gyro by adjusting the electrostatic force generated on the tuning electrodes [28]. This process can also change the resonant frequency of the gyroscope. So, the frequency matching between two gyroscopic modes can be achieved. Under the condition of ignoring the deformation during vibration, the simplified schematic of the tuning electrode is shown in Figure 3. Assuming that the tuning voltage applied on the tuning electrode is *V*, the electrostatic force received by the structure can be expressed as:

(6) $$F_{e}=\frac{S\varepsilon }{2{\left(x-\Delta x\right)}^{2}}V^{2},$$

where *S* is the effective area of the electrode, ε is the permittivity, *x* is the distance between the plates and Δx is the relative displacement. If the initial stiffness of the structure is $k_{0}$, the resultant force *F* can be expressed as:

(7) $$F=k_{0}\left(x+\Delta x\right)-F_{e}.$$

Taking the partial derivative of the resultant force *F* and ignoring the higher order term, the relationship between the structural stiffness and the tuning voltage can be deduced as:

(8) $$k=\frac{\partial F}{\partial \Delta x}=k_{0}-\frac{S\varepsilon }{x^{3}}V^{2}.$$

Since $\omega =\sqrt{k/m}$, the expression of resonant frequency ω under tuning voltage *V* is:

(9) $$\omega =\sqrt{\omega_{0}^{2}-\frac{S\varepsilon }{x^{3}}V^{2}}.$$

It can be seen intuitively from Equation (9) that changing the voltage applied to the tuning electrode can change the resonant frequency of the gyroscopic mode. Therefore, the frequency matching of two gyroscopic modes can be realized by applying appropriate tuning voltage. All the frequency split calibration scheme mentioned in this paper are based on this principle.

### 3.2. Mode Switching Technology

Mode switching is a unique control scheme for gyroscope. Its core idea is to drive the two modes of the gyro alternately [29]. So, the resonant frequency of the two modes can be accurately identified. After one complete switch, the value of frequency split between two gyroscopic modes can be obtained. Based on this value of frequency split, further calibration method can be performed.

**Figure 4:**
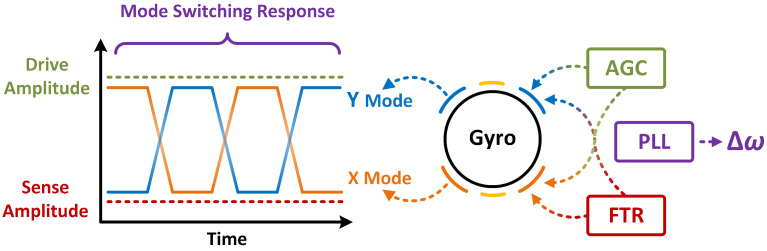
The process of mode switching.

In practical applications, the realization of mode switching requires the cooperation of PLL (Phase Locked Loop) [30], AGC (Automatic Gain Control), and FTR (Force-To-Rebalance) [31] technologies. The PLL is time-division multiplexed to alternately lock on the optimal phase points of two gyroscopic modes. Then the resonant frequencies and frequency split of two gyroscopic modes are obtained. AGC and FTR are used to speed up the rise and decay process of the two modes. AGC can help the amplitude of the drive mode reach the needed value quickly. FTR can make the attenuation of the sensitive mode amplitude fast and stable. These two technologies act alternately on the two gyroscopic modes, thereby shortening the dead time of mode switching. The whole process of mode switching is shown in Figure 4.

By adjusting the parameters of the closed-loop controller, mode switching can quickly complete the identification and calibration of the frequency split. However, the limitations of mode switching are also very obvious. During the mode switching process, the two gyroscopic modes are both in an alternately driven state. Therefore, the detection of the angular rate cannot be completed successfully. So as an offline calibration algorithm, the action time of mode switching is before the normal operation time of gyro. However, the frequency split of the gyroscope will further deteriorate with the extension of the operating time. Therefore, in the long run, the calibration effect of mode switching cannot be particularly ideal.

On the other hand, using mode switching to calibrate frequency split have no limitation on the stability boundary. The frequency split can be calibrated to a small level before the gyro starts to work. This makes the offline calibration scheme using mode switching have good complementarity with the in-run calibration scheme under dual-mode architecture. A hybrid scheme that using both the mode switching and dual-mode architecture will achieve in-run mode matching with no limitation of the stability boundary. However, it is worth noting that mode switching is a technology applied on single-mode gyro scheme. In order to realize the above-mentioned hybrid scheme, the gyro interface system must have the ability to switch between single-mode scheme and dual-mode scheme.

### 3.3. Hybrid Gyro Interface System with Single-Mode and Dual-Mode

**Figure 5:**
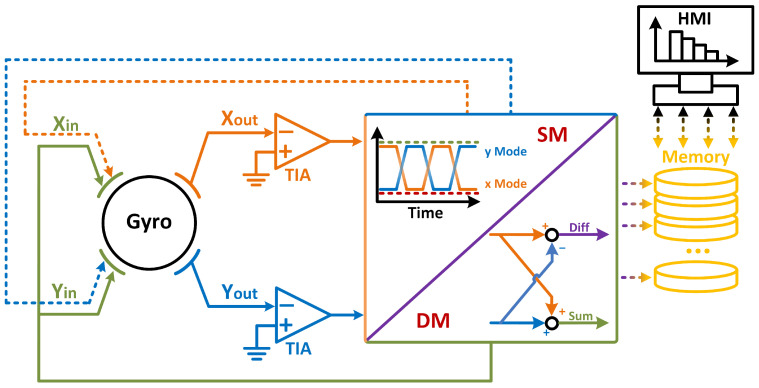
Hybrid gyro interface system with single-mode and dual-mode.

**Figure 6:**
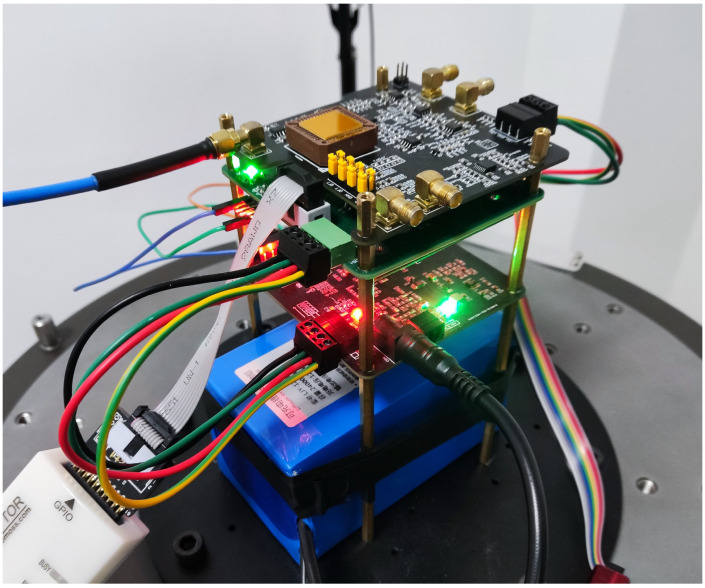
Gyro circuit prototype with hybrid interface system.

Based on the analysis in the previous subsection, this subsection proposes a hybrid gyro interface system with single-mode and dual-mode. The system architecture of this scheme is shown in Figure 5. In this scheme, the change of gyro excitation is realized by adding three program-controlled switches in the interface circuit. The actual gyro interface circuit prototype is shown in Figure 6.

After the gyro interface system is powered on, the mode switching scheme is first applied in the single-mode architecture. At this time, the drive signals of two gyroscopic modes are given by two separate DAC (digital-to-analog converter) modules. The two modes are driven alternately to obtain the value of the frequency split. Then, based on the frequency split, a PI controller is applied to realize the preliminary frequency matching between two gyroscopic modes. After the frequency split is calibrated to a small range, the driving scheme of the gyro is changed to dual-mode architecture by changing the conduction state of the program-controlled switches. Then, the gyro officially enters the operating state. At this time, the two gyroscopic modes are excited by the same DAC module. Another PI controller is used to keep the real part of the differential output signal at zero, so as to achieve the in-run frequency split calibration. The specific effects of this scheme will be introduced in the next section.

## 4. Experimental Results

**Figure 7:**
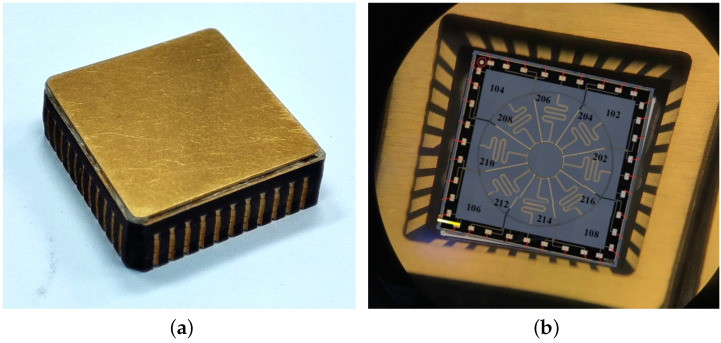
The sample annular MEMS gyro used in experiments. (**a**) The external package of sample gyroscope. (**b**) The internal structure of the sample gyroscope.

In this section, the problem and solution about dual-mode in-run frequency split calibration scheme are verified and analyzed through detailed experiments. All the experimental results are carried out on an annular MEMS gyro with high *Q* value. The sample gyro is shown in Figure 7. The dimensions of gyro are 16 × 16 × 4 mm${}^{3}$. The sensitive electrode structure inside the gyro is connected to the external package through gold wires. In order to reduce the air damping of the gyroscopic sensitive electrodes, the inside of the gyro package is evacuated to vacuum.

### 4.1. The Initial State of the Gyro

**Figure 8:**
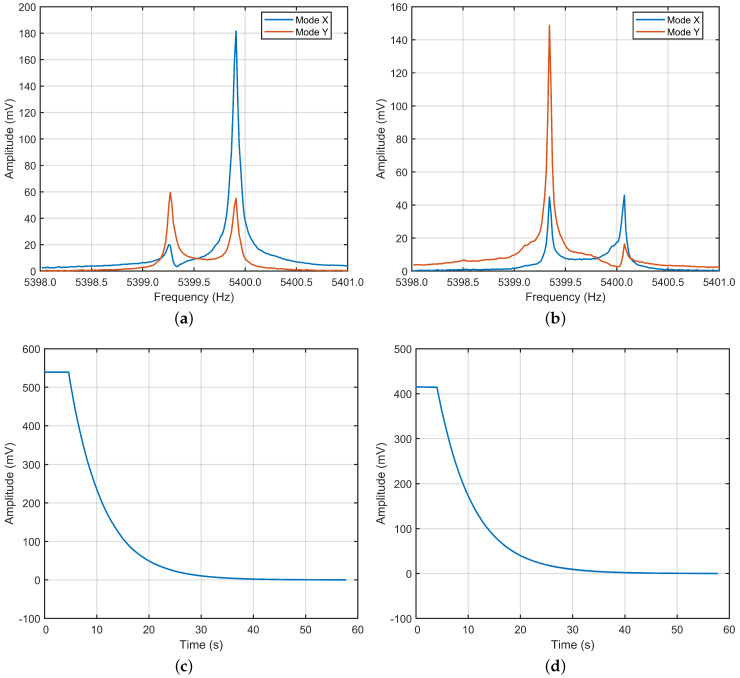
The initial state of the gyro. (**a**) The frequency characteristic of gyro when mode X is excited. (**b**) The frequency characteristic of gyro when mode Y is excited. (**c**) The ring down process of mode X. (**d**) The ring down process of mode Y.

Without applying any calibration algorithm, the two gyroscopic modes are driven separately under single-mode architecture with the same drive signal. The frequency characteristics of the sample gyro are shown in Figure 8a,b. As can be seen from the figure, in the initial state, there is a certain mode mismatch of the gyro. The coupling between the two modes is also large. This means that when one mode is excited, a considerable portion of energy will be coupled to the other mode. As a result, the amplitude peak of the sense mode will appear at the resonance frequencies of the two modes. This will seriously affect the normal angular rate measurement of the gyro.

In this paper, the calculation of the *Q* value is realized by fitting the attenuation curve of the amplitude. The attenuation curves of the two modes’ amplitude are shown in Figure 8c,d. Using the “Curve Fitting Tool” function in MATLAB, the above attenuation curves can be fitted in the form of exponential curves with the expression of f(x)=a∗exp(b∗x). Then, based on the parameters in the fitted curve expression, the *Q* value can be derived by the expression of Q=(π∗ω)/b, where ω is the respective resonant frequencies of two gyroscopic modes. Finally, the *Q* values of the two modes can be calculated as 109.6 k and 115.9 k.

### 4.2. The Stability Boundary While In-Run Mode Matching under Dual-Mode Scheme

#### 4.2.1. The Stability Range of the Mode Matching System

**Figure 9:**
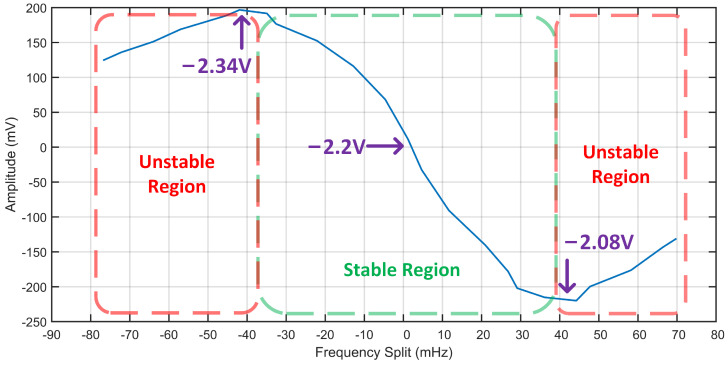
The stability range of the closed-loop control system.

The relationship between frequency split and the real part of difference signal is simulated in Section 2. This section will perform a verification of the stability boundary on the sample gyroscope. By adjusting the tuning voltage, the frequency split is gradually adjusted from −80 mHz to +70 mHz. The amplitude of the difference signal at each sampling point of frequency split is recorded. The experimental results are shown in Figure 9.

As can be seen from the figure, the relationship between the frequency split and the difference signal is highly consistent with the simulation results in Section 2. When the amplitude of difference signal is zero, the value of the frequency split is also approximately zero. At this point, the tuning voltage is −2.2 V. When the frequency split increases to about 40 mHz, the monotonicity of this relationship will change. The corresponding tuning voltages at this point are −2.08 V and −2.34 V, respectively. This is the stability boundaries of the frequency split closed-loop control system. This result not only verifies the rationality of the simulation conditions, but also intuitively illustrates the necessity of the problem studied in this paper in practice.

#### 4.2.2. Verification of Unstable Region

**Figure 10:**
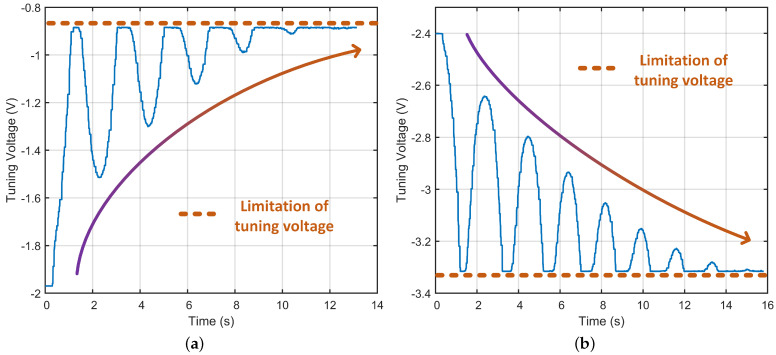
Reverse auto-adjustment of tuning voltage in unstable region. (**a**) Change of tuning voltage when the initial frequency split is greater than 40 mHz. (**b**) Change of tuning voltage when the initial frequency split is greater than −40 mHz.

Based on the results in the previous subsection, this subsection will further analyze the performance of the in-run frequency split calibration when the frequency split exceeds the stability boundary. The results are shown in Figure 10. In Figure 10a, the initial reference tuning voltage is set to −1.97 V, which makes the initial frequency split to be about 65 mHz. In Figure 10b, the initial reference tuning voltage is set to −2.4 V, which makes the initial frequency split to be about −65 mHz. It can be seen that when the initial frequency split exceeds the stability boundary, the suppression of the difference output amplitude will adjust the value of the tuning voltage away from the optimal tuning voltage. Which means the mode mismatch is aggravated. In order to prevent the gyro from being damaged by the change of the tuning voltage in the wrong tuning direction, a limit for the variation range of the tuning voltage is set in the experiment. As shown in the figure, the calibration of frequency split in the unstable regions will eventually adjust the tuning voltage to the upper and lower bounds of the voltage limit. Therefore, the initial frequency split must be calibrated to the stable region before the normal gyro operation if the in-run frequency split calibration under dual-mode architecture is needed. The curved arrow lines in Figure 10 indicate the changing trends of the tuning voltage.

### 4.3. The Pre-Calibration of Frequency Split by Mode Switching

**Figure 11:**
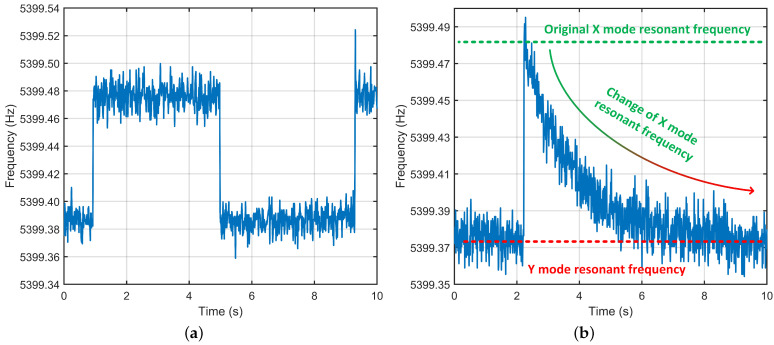
The pre-calibration of frequency split by mode switching. (**a**) The time-division multiplexing of PLL. (**b**) The change of resonant frequency during calibration.

The process of frequency split calibration using mode switching is shown in Figure 11. Figure 11a shows the time-division multiplexing (TDM) of the PLL during the mode switching process. It can be seen that after several cycles of mode switching, the resonant frequencies and the frequency split are obtained. The value of frequency split is updated every two mode switches. This shows that the mode switching inevitably has a hysteresis for mode matching. Based on this frequency split value, the closed-loop control method is applied to the tuning voltage to adjust the resonant frequency of one mode. Finally, the resonant frequencies of the two modes are adjusted to match. The preliminary calibration of frequency split is realized, as shown in Figure 11b. Although the calibration effect of this scheme will demonstrate with the long-time operation of the gyro, the frequency split between two gyroscopic modes is quickly adjusted to a small level. This prepares a prerequisite for the subsequent dual-mode in-run calibration scheme.

### 4.4. The In-Run Calibration of Frequency Split under Enhanced Dual-Mode Scheme

**Figure 12:**
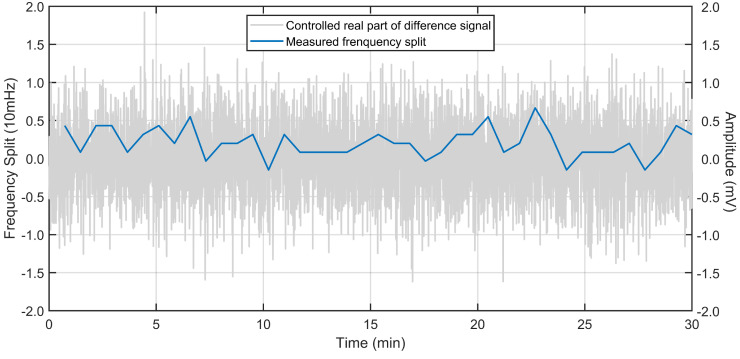
The in-run calibration of frequency split under dual-mode scheme.

After the preliminary calibration of frequency split by mode switching, the in-run calibration of the frequency split under a dual-mode scheme is successfully implemented. The real part of the difference signal is controlled to zero by the PI controller. So, the frequency split between two gyroscopic modes is suppressed in real time. Given that the actual value of frequency split cannot be obtained under this dual-mode scheme, mode switching is applied every minute to verify the calibration effect of the frequency split. This verification experiment is also achieved by changing the conduction state of the program-controlled switch in the gyro interface system. The experimental results are shown in Figure 12.

From Figure 12, it is shown that the real part of the difference signal is stably maintained at zero. The fluctuation range under the action of PI controller is about 2 mV. Correspondingly, the in-run calibration of frequency split under dual-mode scheme shows excellent precision. From the value of each frequency split sampling point, the frequency split in gyro operation is stably suppressed below 5 mHz. This will greatly improve the stability and sensitivity of the gyro.

## 5. Conclusions

In this paper, the stability boundary during in-run mode matching under dual-mode scheme is analyzed through simulations and experiments. When the initial frequency split between gyroscopic modes is too large, the in-run frequency split calibration scheme will fail. The length of the stable region will be affected by the resonant frequency and the *Q* value. High resonant frequency and low *Q* value will widen the stable region of frequency split, but also reduce the sensitivity and rapidity of the calibration. Focusing on this problem, an enhanced solution that combines both the dual-mode scheme and mode switching technology is proposed in this paper. Mode switching is applied to realize the pre-calibration of frequency split before the normal gyro operation. Experimental results show that this hybrid scheme removes the limitation of the stable region while applying the in-run frequency split calibration under dual-mode architecture. The work of this paper is conducive to expanding the application of the dual-mode gyro scheme.

## Data Availability

The data used to support the findings of this study are available from the corresponding author upon request.
